# Intrinsic temporal structure and lagged environmental effects shape the dynamics of airborne microscopic eukaryotes

**DOI:** 10.1128/aem.00286-26

**Published:** 2026-05-05

**Authors:** So-Yeon Jeong, Chi Won Lee, Tae Gwan Kim

**Affiliations:** 1 Department of Microbiology, Pusan National University 34996, Pusan, South Korea; The University of Tennessee Knoxville, Knoxville, Tennessee, USA

**Keywords:** airborne eukaryotes, static analysis, time-series analysis, fungi, protists, metazoa

## Abstract

Airborne microscopic eukaryotes play important ecological roles, yet their temporal dynamics often appear stochastic and difficult to predict using concurrent meteorological variables. We conducted a high-resolution, 3-year survey of airborne fungi, protists, and metazoa in a temperate urban environment using quantitative PCR and amplicon sequencing. Seasonality varied markedly among groups: fungi and metazoa exhibited distinct annual cycles (peaking in late summer and winter, respectively), while protists displayed stochastic fluctuations. Taxonomically, fungi were dominated by soil- and plant-associated taxa (e.g., *Cladosporium*), protists by aquatic and terrestrial taxa (e.g., *Phytophthora*), and metazoa by DNA from mammals and insects. Static (i.e., contemporaneous, non-time lagged) analyses based on correlation and multiple linear regression identified concurrent environmental associations but explained only a limited proportion of the variance (28%, 6%, and 14% for fungi, protists, and metazoa, respectively). In contrast, incorporating time-lagged effects using Granger causality and autoregressive integrated moving average with exogenous variables (ARIMAX) substantially improved explanatory power, accounting for up to 47% of the variance for fungi and ~29% for protists and metazoa. These improvements primarily reflected intrinsic temporal dependence, indicating that antecedent biological states and prior environmental conditions contributed to eukaryotic abundances. Our results suggest that relying solely on concurrent meteorology is insufficient, whereas integrating temporal dependence and lagged environmental effects provides a robust framework for predicting airborne eukaryotic dynamics.

**IMPORTANCE:**

Airborne microscopic eukaryotes influence ecosystems, agriculture, and human health, yet their temporal behavior in the atmosphere remains poorly understood and difficult to predict. Using a 3-year, high-resolution survey, this study shows that different airborne eukaryotic groups follow fundamentally different temporal rules. Airborne fungi, dominated by plant- and soil-associated taxa such as *Cladosporium*, and animal-derived metazoan material exhibit clear and contrasting seasonal cycles, whereas protists, including the plant pathogen *Phytophthora*, fluctuate irregularly. Importantly, models based only on current weather conditions explain little of this variability. By incorporating biological memory and delayed environmental effects, time-series models substantially improve predictability across all groups. These findings demonstrate that airborne eukaryotes respond not only to present conditions but also to prior environmental states, providing a more realistic framework for forecasting bioaerosols relevant to ecosystem connectivity, plant disease spread, and air-quality risk assessment.

## INTRODUCTION

The atmosphere serves as a dynamic medium for diverse microscopic eukaryotes, including fungi, protists, and metazoa (1–3), while also hosting bacteria and archaea (4). These airborne organisms can influence biogeochemical cycling, ecosystem connectivity, and public health (2, 5, 6). Fungi are among the most extensively studied airborne microscopic groups due to their ubiquity as decomposers and their roles as allergens and pathogens (7, 8). Airborne protists (algae and protozoa) have recently received attention for their roles in nutrient cycling and pathogenicity (9–11). Additionally, microscopic metazoa—comprising fragments from insects, mammals, and small invertebrates such as mites and nematodes—represent an important component of bioaerosols (12, 13) and may be responsible for health risks as allergens/pathogens (14).

Airborne microbes originate from heterogeneous sources such as soil, vegetation, and water (2, 5, 15). Once aerosolized, they are exposed to a highly variable atmosphere where meteorological conditions (e.g., temperature and humidity) fluctuate continuously (1, 4). Consequently, airborne microbial communities exhibit pronounced temporal variability in both abundance and community composition (16–18). These temporal patterns arise from the interplay between biological source strength (e.g., growth cycles, sporulation, encystment, and shedding) and environmental factors that regulate aerosolization, atmospheric transport, and deposition (19–21). For instance, rainfall-induced humidity can stimulate fungal growth, resulting in elevated airborne spore concentrations after a physiological delay (22, 23).

Temporal population dynamics are typically analyzed using either contemporaneous correlation-based methods or dynamic time-series analyses. Aerobiological studies frequently employ correlation and regression analyses between microbial metrics and environmental conditions (22, 24, 25) due to their simplicity and compatibility with irregular or limited data sets. However, these static methods treat observations as independent, ignoring the sequential dependence and lagged environmental effects intrinsic to biological systems. For instance, fungal sporulation often follows a growth phase triggered by earlier environmental conditions such as elevated temperature and humidity, generating lagged responses that concurrent methods fail to capture. By contrast, time-series approaches, such as autoregressive integrated moving average (ARIMA) and ARIMA with exogenous variables (ARIMAX), are designed to determine temporal dependence and lagged relationships (26–28). Although time-series methods require long, regularly spaced data sets, they capture ecological memory, whereby present-day abundance reflects antecedent states and prior environmental conditions. While ARIMA models intrinsic temporal patterns by linking current values to past observations, ARIMAX incorporates external predictors to quantify how prior environmental conditions drive current dynamics (29). Therefore, integrating contemporaneous associations with time-series analysis offers a more complete understanding of bioaerosol dynamics than either approach alone.

In this study, we integrated contemporaneous and time-series analyses to characterize the dynamics of airborne fungi, protists, and metazoa in a temperate urban atmosphere. Based on a 3-year, high-resolution survey (April 2019 to May 2022) using quantitative PCR (qPCR) and amplicon sequencing, our specific objectives were to (i) determine seasonal and stochastic variations across the groups; (ii) distinguish immediate environmental drivers from lagged influences; and (iii) evaluate the predictive improvement of dynamic ARIMAX modeling over static multiple linear regression (MLR). By integrating these approaches, we provide a robust framework for predictive aerobiology that explicitly accounts for the temporal dependencies governing microbial fluctuations.

## RESULTS

### Temporal dynamics of airborne eukaryotic groups

**Fig 1:**
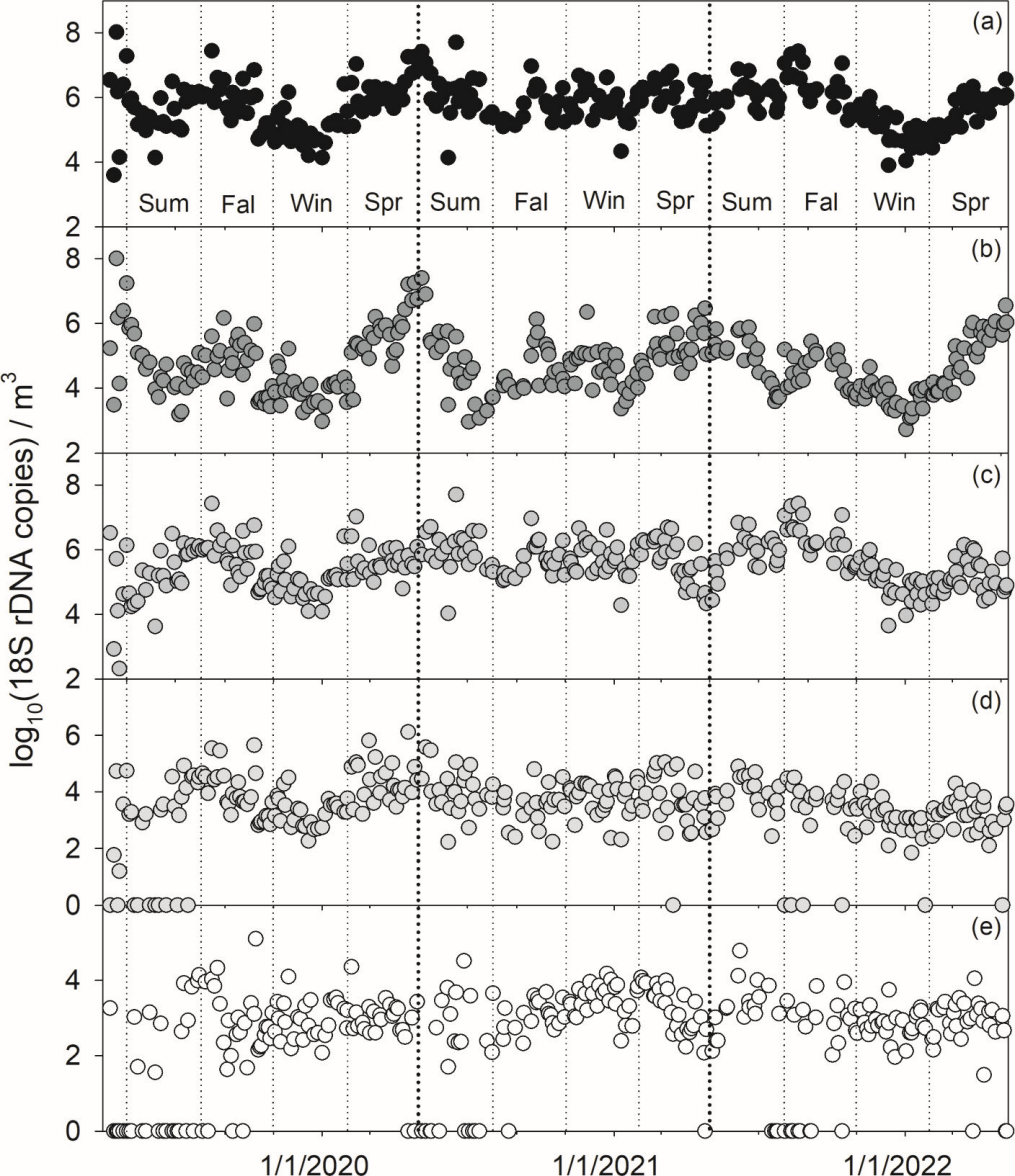
Temporal dynamics of airborne microscopic eukaryote groups over 3 years (April 2019 to May 2022). Time series of 18S rRNA gene abundance are shown for: (**a**) total eukaryotes (all groups combined, expressed as log_10_ copies/m³ of air); (**b**) plant-derived eukaryotic DNA; (**c**) fungi; (**d**) protists; and (**e**) metazoa. Each point represents the abundance in an individual aerosol sample. Vertical dashed lines indicate seasonal transitions, with seasons labeled as spring (Spr), summer (Sum), fall (Fal), and winter (Win).

From April 2019 to May 2022, fungi and plants dominated the 18S rRNA gene sequencing libraries (73.4% ± 24.2% and 24.2% ± 24.8%, respectively; Fig. S2a), whereas protists (1.7% ± 1.5%) and metazoa (0.5% ± 0.7%) were minor components (Fig. S2b). Total eukaryotic abundance (18S rRNA gene copies) averaged 5.75 ± 0.70 log_10_ copies/m³ of air (range: 3.60–8.01; Fig. 1a). Plant DNA (presumably from pollen and plant debris) exhibited a pronounced seasonal pattern, reaching its highest levels in spring (up to 8.01 log_10_ copies/m³) and declining sharply in winter (Fig. 1b). Fungal abundance (5.53 ± 0.74 log_10_ copies/m³) also showed distinct seasonal trends, with concentrations peaking in late summer through autumn and dropping in winter (Fig. 1c), with observed concentrations ranging from 2.32 to 7.69 log_10_ copies/m³. Protists were generally present at much lower concentrations (3.47 ± 1.12 log_10_ copies/m³) and exhibited high temporal variability without a clear seasonal cycle (Fig. 1d). Protist counts fell below the quantitative detection limit in 17 of 317 samples; across the quantifiable samples, abundance varied between 1.10 and 6.11 log_10_ copies/m³. Metazoa were the least abundant group (2.56 ± 1.26 log_10_ copies/m³) and were frequently undetectable (52 samples); when detectable, concentrations ranged from 1.51 to 5.10 log_10_ copies/m³ (Fig. 1e).

### Seasonal variation of airborne eukaryotic groups

**Fig 2:**
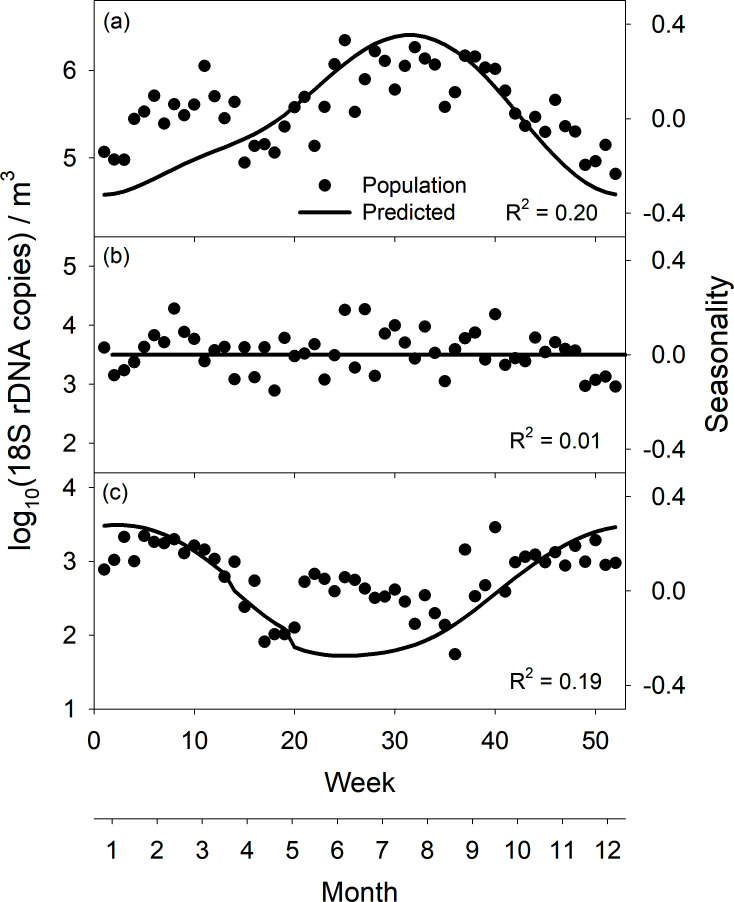
Seasonal variation in airborne fungi (**a**), protists (**b**), and metazoa (**c**). These plots show the average seasonal pattern for each group across the 3-year study. Weekly observations from all years were aligned by week of the year and aggregated to reveal seasonal trends. Seasonal components were extracted using automatic structural time-series decomposition (stR). Symbols (circles) indicate the observed weekly abundances (after aggregation across years), and the solid lines represent the modeled seasonal component for each group (i.e., the recurrent seasonal signal isolated from the time series). The x-axis is labeled by week of year (1–52), and the y-axis is labeled by log_10_ copies/m³ (on the same scale for observed and seasonal values).

The structural time series decomposition revealed clear and distinct seasonal profiles for fungi and metazoa, but not for protists (Fig. 2; Fig. S3 to S5). Spectral analysis further supported these findings: fungi and metazoa showed a strong spectral peak corresponding to a 1-year periodicity, whereas protists displayed no dominant frequency component (Fig. S6). Fungal abundance peaked in late summer (weeks 25–35) and reached a minimum in winter (weeks 50–3), with seasonality explaining ~20% of the variance (Fig. 2a). Metazoan abundance exhibited an almost opposite phase with a peak in winter (weeks 47–5), explaining ~19% of the variance (Fig. 2c). In contrast, protistan abundance showed negligible seasonality with a flat profile throughout the year, accounting for only ~1% of the variance (Fig. 2b).

### Taxonomic composition of airborne eukaryotic groups

Table 1 summarizes the 20 most dominant taxa for each group across all samples. The fungal community was dominated by *Cladosporium* (18.6%), Polyporales (13.3%), Agaricomycetes (11.8%), and Pleosporales (7.0%), with the top 20 taxa accounting for 87.3% of the fungal sequence reads. The protistan community was dominated by *Phytophthora* (32.0%) and Eugregarinorida (17.8%), followed by Colpodida (5.7%); the top 20 taxa comprised 82.3% of the protistan reads. Metazoan sequences were strongly dominated by Mammalia (43.3%), followed by Acari (4.2%), Hymenoptera (2.9%), Hemiptera (2.5%), and Araneae (2.5%), with the top 20 taxa accounting for 75.2% of the metazoan reads.

**TABLE 1:** Relative abundance (%) of the top 20 most dominant taxa in airborne microscopic eukaryotic groups (fungi, protists, and metazoa) over the 3-year period (April 2019 to May 2022)^*a*^

	Fungi		Protista		Metazoa	
	Taxonomy	%	Taxonomy	%	Taxonomy	%
1	*Cladosporium*	18.6	*Phytophthora*	32.0	Mammalia^*b*^	43.3
2	Polyporales^*b*^	13.3	Eugregarinorida^*b*^	17.8	Acari^*b*^	4.2
3	Agaricomycetes^*b*^	11.8	Colpodida^*b*^	5.7	Hymenoptera^*b*^	2.9
4	Pleosporales^*b*^	7.0	Hymenostomatia^*b*^	4.3	Hemiptera^*b*^	2.5
5	Agaricales^*b*^	6.7	Syndiniales^*b*^	2.6	Araneae^*b*^	2.5
6	*Sistotrema*	5.5	Conoidasida^*b*^	2.5	Aves^*b*^	2.2
7	Phanerochaetaceae^*b*^	3.6	*Physarum*	2.1	Coleoptera^*b*^	2.2
8	Boletales^*b*^	3.5	*Heteromita*	2.0	Psocoptera^*b*^	1.9
9	*Aspergillus*	3.1	Peronosporomycetes^*b*^	1.8	Teleostei^*b*^	1.8
10	Russulales^*b*^	2.7	*Protostelium*	1.6	Diptera^*b*^	1.6
11	*Alternaria*	2.5	Spirotrichea^*b*^	1.4	Orthoptera^*b*^	1.4
12	Omphalotaceae^*b*^	1.7	Alveolata^*b*^	1.2	Haplotaxida^*b*^	1.4
13	Corticiaceae^*b*^	1.4	*Rhogostoma*	1.2	Mantodea^*b*^	1.2
14	Leotiomycetes^*b*^	1.3	Platyophryida^*b*^	1.1	Thysanoptera^*b*^	1.2
15	*Coriolopsis*	1.1	Eukaryota^*b*^	1.0	Suberitida^*b*^	1.2
16	Xylariales^*b*^	0.9	Cercozoa^*b*^	1.0	Collembola^*b*^	1.2
17	Aureobasidiaceae^*b*^	0.8	Haptoria^*b*^	0.9	Bilateria^*b*^	1.0
18	Mrakiaceae^*b*^	0.6	Centrohelida^*b*^	0.8	*Lorryia*	0.5
19	Helotiales^*b*^	0.6	*Didymium*	0.7	Heterobranchia^*b*^	0.4
20	Mycosphaerellaceae^*b*^	0.6	*Badhamia*	0.6	Parachela^*b*^	0.4
	Sum	87.3	Sum	82.3	Sum	75.2

^*a*^ Taxonomic assignments were made at the genus level when possible. Values represent the mean relative abundance of each taxon across all samples.

^*b*^ Taxa identified at higher taxonomic ranks (order, class, and family).

### Relationships between airborne eukaryotic groups and environmental factors

**Fig 3:**
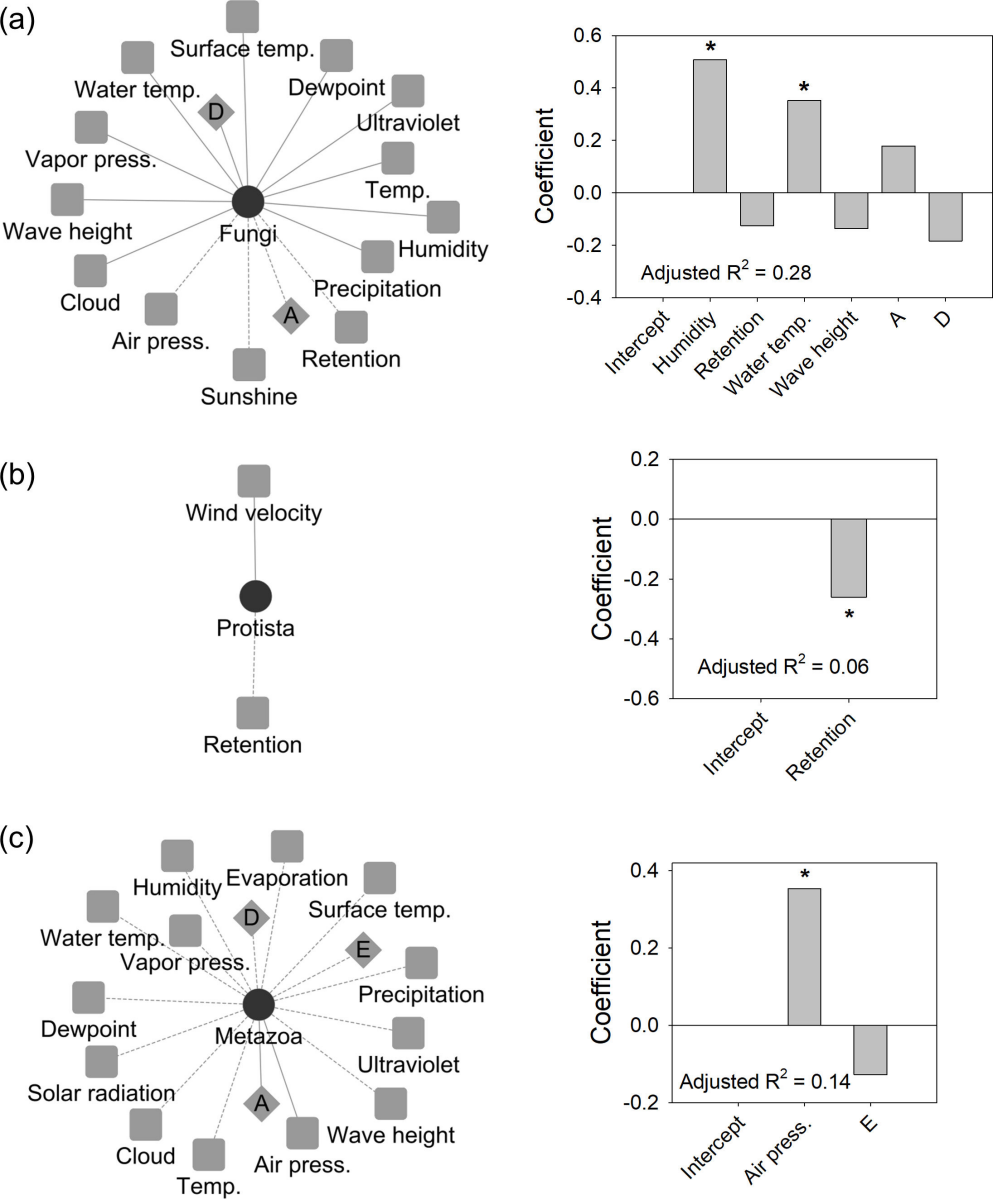
Relationships between airborne eukaryotic groups and environmental factors (concurrent analysis). Panels show results for fungi (**a**), protists (**b**), and metazoa (**c**). Left: Correlation networks depicting significant Pearson correlations (*P* < 0.05) between each group’s abundance and environmental variables. Each node represents either the target eukaryotic group (filled circle), a meteorological variable (square), or an air mass pathway frequency (diamond). Edges indicate significant correlations (solid lines for positive correlations and dashed lines for negative correlations). For clarity, correlations among environmental variables are not shown. Letters A, D, and E represent air mass pathways (A, air masses from the northwest; D, air masses from the south; E, air masses from the east). Right: MLR models identifying key environmental predictors of each group’s abundance. The MLR models were built using variables from the correlation analysis, with highly collinear variables (*r* > 0.85) removed and stepwise selection applied. In each model summary, predictors that remained in the final model are shown; those marked with an asterisk (*) had a statistically significant coefficient (*P* < 0.05). The adjusted *R*² of the final models was 0.28 for fungi, 0.06 for protists, and 0.14 for metazoa, indicating modest explanatory power from concurrent environmental factors.

The correlation network analysis revealed distinct contemporaneous relationships (*P* < 0.05) between abundance and environmental variables for each group (Fig. 3, left). Fungal abundance was associated with multiple environmental factors, showing positive relationships with relative humidity, water temperature (sea surface temperature measured near the sampling site), and precipitation. Metazoan abundance showed predominantly negative associations, specifically with humidity, precipitation, and solar radiation. In contrast, protists exhibited fewer concurrent associations, limited to atmospheric retention (a negative correlation) and wind velocity (a positive correlation).

### Influence of environmental factors on airborne eukaryotic abundance

Stepwise MLR models were constructed for each group using the significant environmental factors identified in the correlation networks (Fig. 3, right). For fungi, the final model explained 28% of the total variance and included the predictors relative humidity, water temperature, atmospheric retention, wave height, and air mass pathways A and D. Among these, humidity and water temperature were significant positive predictors (*P* < 0.05). The metazoan model explained 14% of the variance, with atmospheric pressure identified as the sole significant positive predictor (*P* < 0.05), while pathway E was retained as a marginal negative term. The protist model showed limited explanatory power (adjusted *R*^2^ = 0.06), retaining only atmospheric retention as a significant negative predictor (*P* < 0.05).

### Time-lagged environmental predictors of airborne eukaryotic abundance

**Fig 4:**
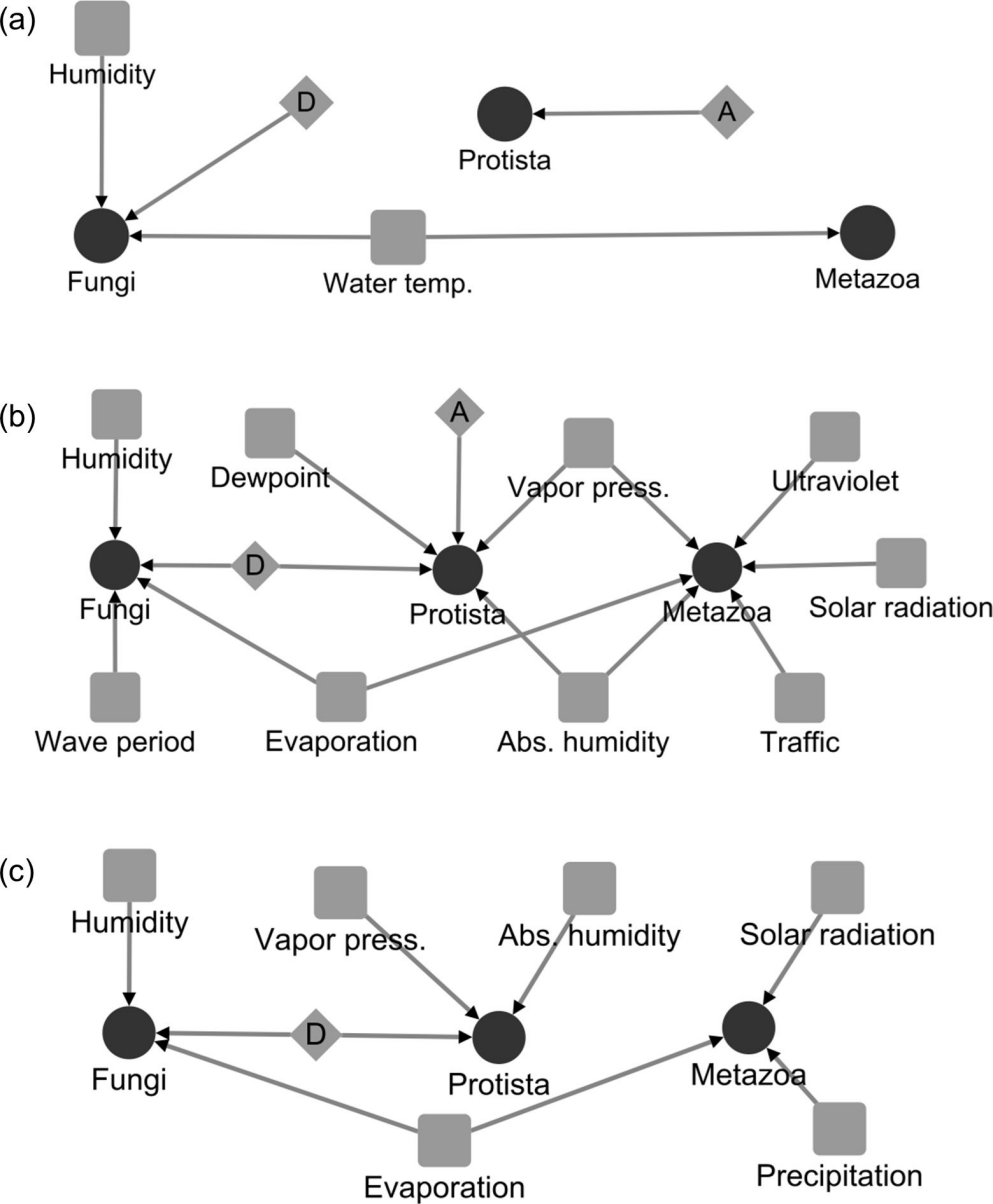
Granger causality networks linking environmental factors to eukaryotic group abundances at different time lags. Networks are shown for a time lag of 1 week (**a**), 2 weeks (**b**), and 3 weeks (**c**). These directed graphs illustrate significant Granger-causal relationships (*P* < 0.05) from environmental variables to the subsequent abundance of fungi, protists, or metazoa. Only causal links from environment to biology are shown (arrows point from squares/diamonds to circles); relationships among environmental variables are omitted for clarity. Node shapes are as defined in Fig. 3. Arrows denote the direction of influence (e.g., an arrow from humidity to fungi at lag 1 means humidity levels 1 week prior have a predictive effect on fungal abundance).

The Granger causality analysis identified time-lagged predictors (1–3-week lags; *P* < 0.05) for each microbial group (Fig. 4), although the test does not establish whether the associations are positive or negative. At a 1-week lag (Fig. 4a), the Granger networks were relatively simple: fungal abundance was predicted by humidity, water temperature, and air mass pathway D; protists by pathway A; and metazoa by water temperature. Network complexity increased at a 2-week lag (Fig. 4b), with fungi linked to four predictors (e.g., wave period, evaporation, and humidity); protists to five factors (e.g., dew point and vapor pressure); and metazoa to six predictors (e.g., evaporation, vapor pressure, and solar radiation). At a 3-week lag (Fig. 4c), the networks simplified, but persistent drivers remained: humidity, evaporation, and pathway D for fungi; vapor pressure, absolute humidity, and pathway D for protists; and precipitation, evaporation, and solar radiation for metazoa.

### Temporal dependence and improved prediction of airborne eukaryotic abundance

**Fig 5:**
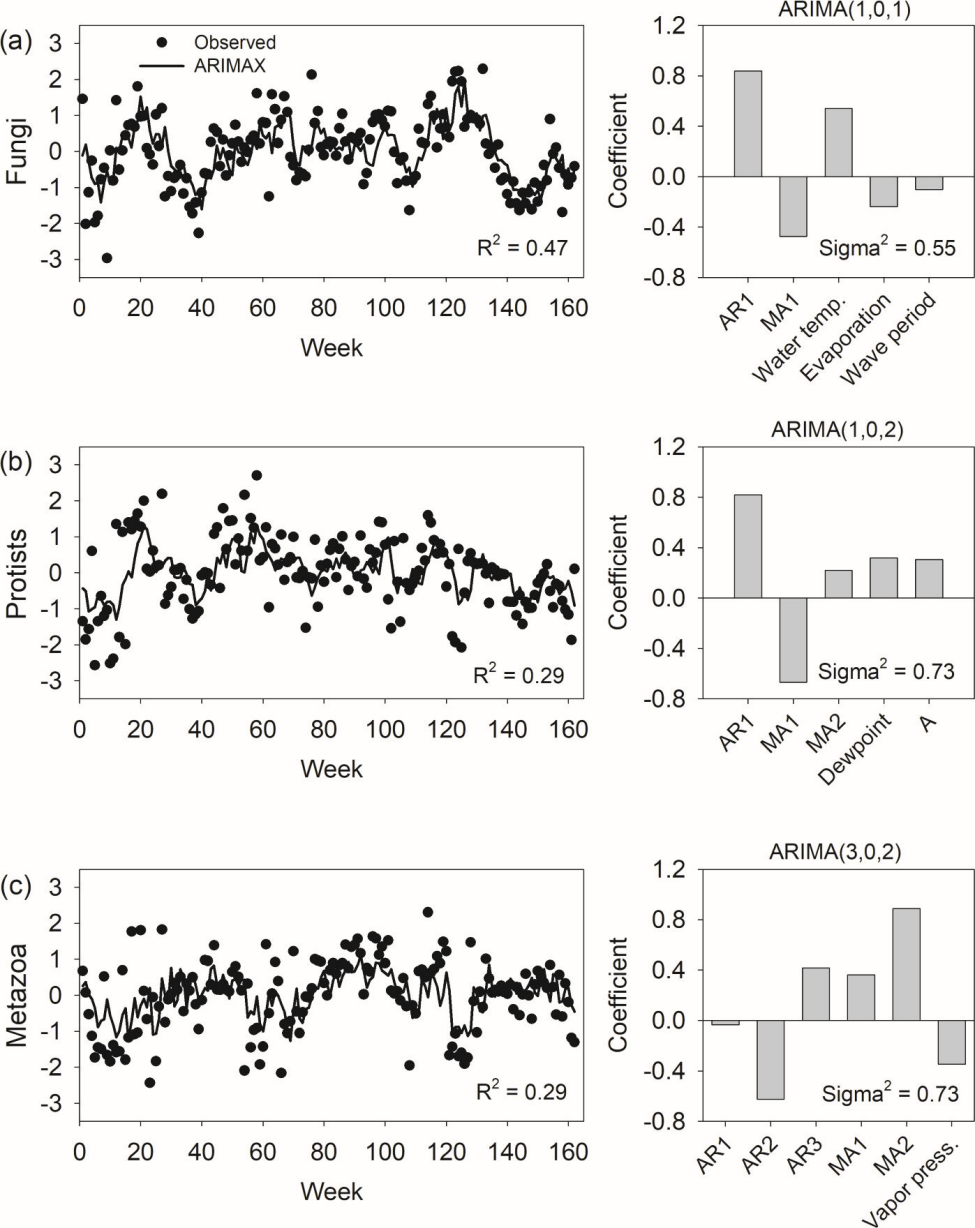
ARIMAX model results for airborne fungi (**a**), protists (**b**), and metazoa (**c**). ARIMAX models were developed for each microbial group, integrating the group’s internal time-series dynamics with lagged environmental predictors identified via Granger causality. To avoid multicollinearity, highly inter-correlated environmental variables (Pearson *r* > 0.85) were excluded from consideration, and model selection was based on minimizing AICc. Left panels: observed weekly abundances (points) versus ARIMAX model fitted values (lines) for each group. A close match between points and line indicates that the model is capturing the temporal fluctuations well. Right panels: summary of each selected ARIMAX model’s structure and parameters. The ARIMA component of each model is given in parentheses as (p,d,q) representing the number of autoregressive terms, the degree of differencing, and the number of moving average terms, respectively. For example, ARIMA(1,0,1) indicates one AR term and one MA term with no differencing. The specific exogenous (environmental) variables included in each model are listed along with their qualitative effect (+ or –) on abundance. For instance, the fungal ARIMAX model (1,0,1) included water temperature (+), evaporation (–), and wave period (–) as exogenous inputs. These models demonstrate the contribution of both past internal states and past environmental conditions to predicting current microbial abundances.

The ARIMAX models were constructed by evaluating all combinations of significant lagged drivers identified via Granger causality (Fig. 5). For fungi, the best model utilized an ARIMA(1,0,1) structure capturing strong temporal dependency (AR1 = 0.8370; MA1 = –0.4746) and three exogenous variables: water temperature (positive, 0.5415), evaporation (negative, –0.2396), and wave period (negative, –0.1025). This model explained approximately 47% of the variance (pseudo-*R*^2^), with a residual variance (σ²) of 0.55, an Akaike Information Criterion corrected (AICc) of 369.52, and a root mean square error (RMSE)of 0.727. For protists, the optimal ARIMAX model had an ARIMA(1,0,2) base (Fig. 5b), reflecting considerable temporal structure (AR1 = 0.8185; MA1 = –0.6708, MA2 = 0.2194), and incorporated dew point (0.3175) and air mass pathway A (0.3041) as positive predictors. This model explained ~29% of the variance (σ² =0.7252; AICc = 415.68; RMSE = 0.838). For metazoa, the selected ARIMAX model required a complex ARIMA(3,0,2) structure (Fig. 5c) with prolonged internal memory (AR1 = –0.0337, AR2 = –0.6242, AR3 = 0.4173; MA1 = 0.3620, MA2 = 0.8885). Vapor pressure emerged as the sole significant exogenous predictor (negative, –0.3464), and the model explained ~29% of the variance (σ² =0.7349; AICc = 419.36; RMSE = 0.841).

## DISCUSSION

Airborne microbial eukaryotes are ecologically diverse and include taxa relevant to health, yet their temporal dynamics and environmental drivers remain poorly characterized, particularly over multi-year timescales. Understanding how these communities fluctuate and what governs their variability is a fundamental question in aerobiology with implications for both environmental monitoring and public health. To address this gap, we investigated the long-term population dynamics of airborne eukaryotes using a 3-year, high-resolution monitoring data set. Over the 3-year study period, airborne eukaryotic communities were consistently dominated by fungi (5.53 ± 0.74 log_10_ copies/m³), followed by protists (3.47 ± 1.12 log_10_ copies/m³) and metazoa (2.56 ± 1.26 log_10_ copies/m³). Structural time-series decomposition and spectral analysis confirmed pronounced annual periodicity for fungi and metazoa, whereas protists exhibited stochastic fluctuations without a regular cycle (Fig. 2; Fig. S6). Fungal abundance peaked in late summer to early autumn and reached a minimum in winter, with the seasonal component explaining approximately 20% of the variance. Metazoan abundance showed a distinct winter peak, with seasonal effects accounting for about 19% of the variance. These contrasting seasonal patterns likely reflect the combined influence of biological source dynamics and season-specific atmospheric conditions. Warm and humid conditions during summer and early autumn favor fungal growth and sporulation, leading to enhanced spore release, whereas winter conditions are less conducive to fungal proliferation. In contrast, the winter peak in metazoa may be associated with seasonal life-cycle processes (e.g., insect senescence and increased production of dry biological debris) combined with meteorological conditions that promote particle retention. Cold, dry winters are often characterized by stable high-pressure systems that suppress atmospheric mixing, allowing fine animal- and insect-derived particulates to accumulate near the surface. Protists, by comparison, showed minimal seasonal structure, with the seasonal component explaining only ~1% of the variance. This lack of predictability suggests that protist aerosolization is governed primarily by sporadic and transient events—such as episodic blooms followed by wind- or spray-mediated release—rather than by recurring seasonal drivers. Consistently, previous studies have shown that some protists can be intermittently transferred to the atmosphere via marine spray and wind-driven dispersal from aquatic environments (30, 31). However, to our knowledge, no previous study has characterized the temporal dynamics of airborne protists at comparable duration and resolution, limiting direct quantitative comparisons. Our 3-year, high-resolution survey therefore provides one of the first systematic baselines for understanding the irregular temporal behavior of airborne protists. Overall, these results indicate that the aerosolization of fungi and metazoa is partly shaped by regular seasonal forcing, whereas protistan dynamics are dominated by irregular, event-driven processes.

The taxonomic composition highlights distinct ecological sources and dispersal pathways across groups (Table 1). Fungal communities were dominated by taxa typically associated with terrestrial ecosystems, including *Cladosporium* (18.6%), Polyporales (13.3%), and Agaricomycetes (11.8%). For example, *Cladosporium* is a ubiquitous genus of leaf- and soil-inhabiting fungi known to produce abundant airborne conidia (32, 33). The prominence of these taxa suggests that soils and plants were the primary emission sources of fungal spores, aligning with the finding that airborne fungal loads are strongly influenced by local vegetation biomass and plant litter (17, 34). Protistan communities were notably dominated by *Phytophthora* spp. (32.0%), a destructive oomycete plant pathogen (35, 36). They produce resilient spores that function as survival structures and dispersal units (37). Their high abundance implies a significant potential for aerial dispersal. In addition, the protistan assemblage included taxa indicative of diverse source environments and lifestyles: insect-associated gregarine protozoa (Eugregarinorida, often parasites of invertebrates), free-living soil ciliates (Colpodida), freshwater ciliates (Hymenostomatia), and marine parasites (Syndiniales). This heterogeneity points to multiple aerosolization pathways ranging from soil dust resuspension to freshwater splash and sea spray, providing a logical explanation for the stochasticity observed in the protist time series. While specific taxa like *Phytophthora* may be seasonal, the composite signal integrates temporally asynchronous inputs from ecologically distinct sources, effectively masking individual patterns. Metazoan sequences were largely classified as Mammalia (43.3%), likely reflecting shed biological material (e.g., skin and hair) from terrestrial vertebrates, followed by arthropods—particularly mites (Acari) and insects (e.g., Hymenoptera and Hemiptera), including microscopic taxa. This composition is consistent with the mechanical suspension of biological debris via wind or physical disturbance. The dominance of terrestrial detritus supports our finding that metazoan abundance peaks in winter, when atmospheric stability favors the retention of suspended particulates near the surface.

To assess immediate environmental drivers, we examined concurrent relationships using correlation networks and MLR models (Fig. 3). For fungi, the MLR model explained 28% of the variance, identifying relative humidity and water temperature as significant positive predictors. The seasonal peak of fungal abundance in late summer to early autumn and its positive association with humidity and temperature are consistent with established patterns reported across temperate regions (22, 23). For example, warm air temperatures are positively correlated with high densities of dominant airborne taxa such as *Cladosporium* and *Alternaria* (32, 38), and post-rainfall moisture conditions stimulate fungal proliferation and subsequent spore release (22, 23). Thus, the concurrent (static) environmental associations identified in our study largely corroborate existing knowledge. For metazoa, the best MLR model retained only atmospheric pressure as a significant positive predictor, explaining 14% of the variance. Metazoan abundance and air pressure both peaked in winter (Fig. S10), suggesting higher metazoan DNA concentrations under high-pressure conditions. Such systems are typically associated with subsiding air and weaker winds, which increase atmospheric stability and can promote near-surface particle accumulation by limiting dispersion and dilution (39–41). Moreover, negative correlations with humidity and precipitation (higher in summer) are consistent with enhanced wet deposition and removal of particles during moist conditions. These findings are less extensively documented in the aerobiological literature, as most bioaerosol studies focus on fungal and bacterial communities. They also underscore that atmospheric stability is a key determinant of the near-surface retention and concentration of coarse biological particles. In contrast, protists exhibited few significant concurrent associations with measured environmental variables. Atmospheric retention (an index of air stagnation) was the only variable retained in the protist MLR model, explaining just 6% of the variance. More dynamic air movement (lower retention) may promote protist aerosolization and transport—for example, by resuspending cells or cysts from soil and water surfaces and enhancing dispersal (42, 43). However, the limited explanatory power, together with the apparent temporal irregularity of protist abundance, suggests that protists were supplied from multiple heterogeneous sources and were only weakly coupled to meteorological variation. Overall, these static analyses suggest that fungal and metazoan dynamics are more strongly associated with prevailing environmental conditions, whereas protistan dynamics are less well explained by contemporaneous predictors.

To identify lagged environmental influences, we next performed Granger causality analysis (Fig. 4). For fungi, humidity and air mass pathway D showed persistent Granger-causal links across 1–3-week lags, indicating that antecedent moisture conditions and repeated southeast-origin air-mass incursions provided predictive information about subsequent fungal abundance. Such persistence is consistent with short-term memory in fungal aerosols, whereby humid periods can promote growth and sporulation that elevate spore release for several weeks. Similarly, the predictive influence of pathway D likely reflects marine moisture transport or wet-season meteorology that promotes sustained fungal proliferation. Protists exhibited more intermittent lagged associations. Protist abundance was linked to air mass pathway A at a 1-week lag, whereas moisture-related variables (dew point, vapor pressure, and absolute humidity) and pathways A and D emerged primarily at 2–3-week lags. These patterns suggest that sustained moisture conditions or discrete transport events can precede transient increases in protist aerosols. For metazoa, water temperature was Granger-causal at a 1-week lag, and evaporation, precipitation, solar radiation, and vapor pressure were significant predictors at 2–3-week lags. These delayed associations are consistent with lagged production and removal processes—for example, dry periods may promote desiccation and fragmentation of biological material that subsequently becomes airborne, whereas precipitation can enhance removal through wet deposition. Overall, the Granger results indicate that prior environmental conditions contain predictive information about later airborne eukaryote abundances, highlighting lagged effects (i.e., ecological memory) that are not captured by contemporaneous correlation-based analyses.

The importance of intrinsic temporal dependence and delayed environmental effects was further supported by the ARIMAX models (Fig. 5). Across all three groups, ARIMAX substantially improved model performance relative to static MLR, increasing explained variance by roughly two- to fivefold. This pattern indicates that a considerable fraction of airborne eukaryote variability reflects autocorrelation and time-lagged processes rather than purely instantaneous responses to contemporaneous conditions. In addition, ARIMAX selected predictors that differed from those highlighted by MLR in some cases, consistent with the idea that drivers of short-term fluctuations may differ from those apparent in concurrent analyses. For fungi, the best ARIMAX model combining strong autoregressive and moving-average structure with lagged environmental predictors (water temperature positive; evaporation and wave period negative) explained ~47% of the variance, compared with 28% in the MLR model. For protists, the contrast was even larger: the best ARIMAX model (including lagged dew point and air mass pathway A, together with AR and MA terms) explained ~29% of variance versus ~6% for MLR, suggesting that protist dynamics contain meaningful temporal structure despite appearing irregular in static analyses. More generally, these results reflect short-term persistence in protist abundance and lagged sensitivity to antecedent moisture and transport conditions. The best metazoan ARIMAX models also improved explanatory power (~29% vs 14% for MLR), with vapor pressure retained as an exogenous predictor and a higher-order ARIMA structure indicating stronger multi-week dependence. Notably, fungi were best described by a low-order ARIMA structure (1,0,1), whereas metazoa required a higher-order structure (3,0,2), implying shorter temporal dependence for fungal spores and longer persistence for metazoan particles. Biologically, this difference could mean that fungal spore concentrations respond relatively quickly and do not depend strongly on values from many weeks prior (consistent with short generation times and immediate release to favorable conditions), whereas metazoan particle levels might accumulate or be influenced by events from several weeks in the past. Taken together, the ARIMAX results demonstrate that models ignoring temporal structure systematically underestimate explanatory power. The critical advance lies not in identifying new environmental predictors *per se*, but in three complementary insights that static methods cannot provide: (i) quantifying the degree to which current bioaerosol levels carry information from their own recent history; (ii) revealing time-lagged environmental effects invisible in concurrent analyses; and (iii) demonstrating that much of the variance left unexplained by static models is attributable to delayed biological and environmental processes rather than to unmeasured factors.

By integrating molecular quantification with coupled static and time-series frameworks, this study provides complementary insights that neither approach alone can fully capture. The static analyses identified contemporaneous meteorological associations, whereas the time-series analyses revealed delayed responses and intrinsic persistence that would otherwise go undetected. From an applied perspective, forecasting or managing bioaerosols may therefore benefit from incorporating not only real-time weather data but also prior environmental conditions, as time-series modeling explicitly captures how processes unfolding over the preceding weeks shape current bioaerosol levels.

### Conclusions

This study provides a comprehensive, multi-year characterization of airborne microscopic eukaryotes in a temperate urban environment by integrating molecular quantification with advanced time-series modeling. The major findings and their implications are summarized below.

#### Seasonal dynamics

Fungi and metazoa exhibited distinct and predictable seasonal patterns, whereas protists did not. Fungal abundance peaked in late summer to early autumn, driven by warm, humid conditions conducive to growth and sporulation. Conversely, metazoan abundance peaked in winter, likely reflecting atmospheric stagnation and high-pressure systems that favor the accumulation of suspended particulates. Protists showed no consistent seasonal signal, indicating that their aerosolization is driven by irregular, episodic events rather than recurring seasonal forces.

#### Ecological sources

Taxonomic composition of each group reflected different ecological sources and dispersal pathways. Fungal assemblages were dominated by ubiquitous terrestrial taxa, including plant- and soil-associated fungi such as *Cladosporium*. Protistan assemblages were dominated by *Phytophthora* and included taxa originating from soil, freshwater, and marine environments, indicating heterogeneous source regions and multiple aerosolization routes. Metazoan material was largely derived from terrestrial animals and arthropods (e.g., mammalian debris, mites, and insect fragments).

#### Immediate environmental associations

Correlation networks and MLR identified several contemporaneous relationships between environmental conditions and eukaryotic abundances. Fungal levels were positively associated with relative humidity and temperature, consistent with their summertime peaks, while metazoan abundance was linked to higher atmospheric pressure and inversely related to wind and moisture, aligning with winter stagnation conditions. However, MLR models explained only a modest fraction of the variance (approximately 28% for fungi, 14% for metazoa, and 6% for protists), indicating that contemporaneous conditions alone provide an incomplete explanation of airborne eukaryote variability, particularly for protists.

#### Lagged effects and improved modeling

Granger causality analyses showed that environmental conditions in preceding weeks (e.g., humidity, air-mass origin, and dew point) contained predictive information about later changes in abundance. ARIMAX models that integrated lagged predictors and temporal dependence improved performance markedly, explaining ~47% of the fungal variance and ~29% for both protists and metazoa. Together, these results suggest that airborne microbial dynamics reflect both delayed environmental responses and intrinsic temporal dependence, with pronounced autocorrelation in the abundance time series.

#### Implications

Together, these findings show that combining static and dynamic approaches yields a more complete understanding of airborne eukaryote dynamics. By explicitly representing temporal dependence and lagged effects, time-series modeling improves the interpretation of past variability and supports the forecasting of bioaerosol dynamics, with relevance to air-quality monitoring, risk assessment (e.g., allergen exposure and pathogen dispersal potential), and predictive tools for environmental and public health applications.

## MATERIALS AND METHODS

### Sampling site

This study was conducted in Busan, Republic of Korea, a mid-latitude temperate region characterized by four distinct seasons, with hot, wet summers and cold, dry winters. The sampling site was located at the Biology Building of Pusan National University (35°23'N, 129°08'E; altitude 79 m; Fig. S1). Busan is a metropolitan city covering approximately 770 km² with a population of around 3.3 million. The annual average temperature and total precipitation during the study period were 15.5°C and 1,676 mm, respectively, according to the Korea Meteorological Administration (KMA; https://www.kma.go.kr).

### Aerosol collection

Two identical aerosol sampling devices were assembled as previously described (44). Each device consisted of a filter-holding cup connected to a vacuum pump. The filter cups were sterilized and covered with a 1-mm nylon mesh to exclude macroorganisms.

During deployment, the two devices were placed horizontally about 0.5 m apart. Aerosols were collected onto polyethersulfone membrane filters (47 mm diameter, 0.2 µm pore size; SciLab Korea Co., Ltd., Seoul, Korea). Blank control filters (unused filters handled identically) confirmed the absence of microbial contamination. Aerosol sampling was conducted regularly from April 2019 to May 2022. Each sampling event lasted 3 hours at a flow rate of 100 L/min, collecting a total of 36 m³ of air per event (18 m³ per device). In total, 317 aerosol samples were collected over the study period.

### DNA extraction

After each sampling, filters were aseptically cut into pieces and transferred to 2 mL screw-cap bead tubes (NucleoSpin Soil kit, Macherey-Nagel, Düren, Germany) and stored at –25°C for 1–3 months until DNA extraction. DNA was extracted using the NucleoSpin Soil kit protocol with minor modifications: an additional sonication step was included by immersing tubes in a 10 L ultrasonic bath (30 min at 60°C, 40 kHz; Daihan Scientific Co., Ltd., Wonju, Korea) prior to chemical lysis to improve cell disruption. Genomic DNA was eluted in 50 µL of elution buffer and quantified using a NanoDrop 2000 spectrophotometer (Thermo Fisher Scientific, Waltham, MA, USA).

### Quantification of the 18S rRNA gene

Eukaryotic abundance in each sample was quantified by qPCR targeting the 18S rRNA gene, with the primer pair 1132F (5′-AYTTRAAGDAATTGACGG-3′) and 1423R (5′-GGGCATYWCDGACCTGTT-3′) (45). *Aspergillus*-derived DNA served as the qPCR standard (46). Each 20 μL qPCR reaction contained 2 μL of 10 × PCR buffer (Cancerrop Inc., Seoul, Korea), 2 µL of 2 mM dNTPs, 0.4 µL of forward primer (10 µM), 0.8 µL of reverse primer (10 µM), 1 µL of 5 × Invitrogen SYBR (Thermo Fisher Scientific), 2 U of Taq DNA polymerase (Cancerrop), and 1 µL of template DNA. Negative control reactions (no-template controls) used sterile water in place of DNA. The amplification program consisted of an initial denaturation step (95°C for 2 min) followed by 40 cycles of 95°C for 30 s, 52°C for 30 s, and 72°C for 30 s (Applied Biosystems StepOnePlus real-time PCR). Fluorescence was read at 82°C at the end of each cycle to monitor amplification.

### rRNA gene amplicon sequencing

Taxonomic composition was analyzed by Illumina MiSeq high-throughput sequencing of 18S rRNA gene amplicons. Multiplex MiSeq sequencing was performed using a composite primer pair based on E1132F/E1423R, as described by Jeong S-Y, Choi J-Y (47). PCR products were purified (FavorPrep PCR purification kit, Favorgen Corp., Taiwan) and quantified, then subjected to a second round of PCR to attach Illumina adapters and indices. The final indexed PCR products were purified again, pooled in equimolar concentrations, and sequenced on an Illumina MiSeq platform by Macrogen Inc. (Seoul, Korea).

### Environmental data and air mass trajectories

Meteorological data (e.g., temperature, humidity, precipitation, etc.) for the sampling period were obtained from KMA (Fig. S7 to S10). Daily measurements from three nearby weather stations were averaged to represent local atmospheric conditions. Air mass movement patterns were analyzed using NOAA’s Hybrid Single Particle Lagrangian Integrated Trajectory (HYSPLIT) model (48). For each sampling day, a 72-hour backward air mass trajectory ending at the sampling site (arrival height of 10 m above ground) was computed using the NCEP/NCAR Reanalysis data set. Based on these trajectories, we identified predominant air mass pathways influencing the site. Six pathway categories (A–F) were defined through visual inspection of trajectory plots (Fig. S1) (49).

### Sequence processing and taxonomic assignment

The sequencing depth of the 18S rRNA gene amplicon libraries averaged 25,300 ± 19,333 reads per sample. Raw sequencing reads were processed using the QIIME 2 pipeline (https://qiime2.org). Sequences were quality-filtered and denoised to identify 6,756 amplicon sequence variants (ASVs). These ASVs were taxonomically assigned using the SILVA 132 reference database for small subunit rRNA, formatted for QIIME 2 compatibility. Any ASVs classified as Bacteria, Archaea, or organellar DNA (chloroplasts/mitochondria) were removed prior to downstream analysis. All remaining ASVs were aggregated at the genus level when possible. A total of 286 out of 317 samples yielded successful sequencing libraries (the remaining samples did not amplify sufficiently).

### Data analysis

All data analyses were conducted in R (version 4.3.0; R Core Team). The abundances of fungi, plants, protists, and metazoa were quantified for each sample by integrating the qPCR-derived total 18S rRNA gene copy number with the relative abundances from sequencing. Specifically, for each sample, we multiplied the relative abundance of each group by the total 18S rRNA gene copies to estimate absolute abundance. For 31 samples for which sequencing data were not available, group abundances were estimated by interpolation. Of these, 23 samples (collected between 13 October 2019 and 21 January 2020) were imputed using the average group abundance of matching 10-day calendar intervals in the subsequent years (2020–2022) to reflect seasonal patterns. The remaining eight missing samples were interpolated linearly from neighboring values. All abundance data were log_10_(*x* + 1)–transformed prior to analysis.

The data set was aggregated into a weekly time series by averaging observations within each week to analyze temporal trends. For weeks with missing observations, values were imputed using a weighted moving average of the preceding 3 weeks: *x*_*t*_ = 1/6 × *x*_*t*-3_ + 1/3 × *x*_*t*-2_ + 1/2 × *x*_*t*-1_. Outliers in the time series (weeks with abnormally high or low values) were identified using boxplot statistics and corrected by the same moving-average imputation method. To detect periodic cycles in eukaryote abundances, we performed spectral analysis on the weekly time series using the spectrum function to estimate spectral density and identify dominant frequency components. Seasonal dynamics were further examined using automatic structural time series decomposition (the AutoSTR function in the stR package), which partitions the time series into trend, seasonal, and stochastic components.

Correlation-based network analysis was used to explore contemporaneous associations between eukaryotic group abundances and environmental variables (Table S1). Pearson correlation coefficients were calculated for all pairwise combinations of biological and environmental variables across the weekly data set. Statistically significant correlations (*P* < 0.05) were visualized as networks using Cytoscape (version 3.8.2). For clarity, correlations among environmental variables themselves were not displayed in these networks—only links between eukaryotic abundance and environmental factors were considered. MLR was then applied to identify key environmental predictors for the abundance of each eukaryotic group. Candidate predictor variables were initially chosen from those that showed significant correlations with the target group in the correlation analysis. To reduce multicollinearity, if two or more environmental variables were highly correlated with each other (Pearson *r* > 0.80), only one representative from such a cluster was retained for regression analysis. We employed a stepwise model selection procedure (using the stepAIC function in the MASS package) (50) to determine the most parsimonious set of predictors for each group’s abundance. The final selected MLR models were checked for statistical significance and for multicollinearity (ensuring variance inflation factor <5 for all predictors).

To infer potential delayed (lagged) relationships between environmental factors and microbial abundance, we conducted Granger causality analyses on the weekly time series. Prior to Granger testing, each time series was assessed for stationarity using the Augmented Dickey-Fuller test (in the tseries package) (51). If needed, first- or second-order differencing was applied to achieve stationarity. We then performed Granger causality tests (using the grangertest function in the lmtest package) (28, 52) for each pair of environmental variable (as predictor) and microbial group (as response) at time lags of 1, 2, and 3 weeks (Table S1). Significant Granger-causal relationships (*P* < 0.05) were used to construct directed networks illustrating which environmental variables provided predictive information about subsequent changes in microbial abundance. In these Granger causality networks, arrows were drawn from environmental factors to eukaryotic groups when the past values of the environmental factor significantly improved the prediction of the group’s future values. As with the correlation networks, connections among environmental variables were excluded for simplicity. To build predictive time-series models that incorporate both intrinsic temporal patterns and external environmental influences, we developed ARIMAX models (53). Prior to modeling, the time series for each group and each candidate environmental variable were scaled to have a mean of 0 and unit variance, to improve numerical stability and comparability of coefficients. ARIMAX model fitting was performed using the forecast package. We first used the auto.arima function to identify a suitable ARIMA model for each group’s abundance time series, and then exogenous environmental variables were incorporated into the model. The pool of candidate exogenous predictors for each group consisted of those environmental factors that showed significant Granger causal relationships at lags 1–3 weeks for that group. We evaluated all possible combinations of these candidate exogenous variables in an ARIMAX framework, selecting the model with the lowest AICc as the final model.

## Data Availability

All sequence data have been deposited in the DNA Data Bank of Japan Sequence Read Archive (https://www.ddbj.nig.ac.jp/dra) under accession number DRA017052. The data underlying this article will be shared on request to the corresponding author.

## SUPPLEMENTAL MATERIAL

Supplemental material aem.00286-26-s0001.docx Table S1; Fig. S1 to S10.

ASM does not own the copyrights to Supplemental Material that may be linked to, or accessed through, an article. The authors have granted ASM a non-exclusive, world-wide license to publish the Supplemental Material files. Please contact the corresponding author directly for reuse.
