# Supplementary material for: Intrinsic temporal structure and lagged environmental effects shape the dynamics of airborne microscopic eukaryotes
Source: Appl Environ Microbiol. 2026 May 5;92(6):e00286-26. doi: 10.1128/aem.00286-26 (PMC13274392; doi:10.1128/aem.00286-26)
Supplement: Supplemental material — Table S1; Fig. S1 to S10. [file aem.00286-26-s0001.docx]

Table S1. Environmental factors used in the correlation, multiple linear regression (MLR), and autoregressive integrated moving average with exogenous variables (ARIMAX) analyses for each airborne eukaryotic group (fungi, protists, and metazoa). For the correlation and Granger causality analyses, all variables within each category were initially screened as candidate predictors. For the MLR and ARIMAX models, only variables retained in the final selected models are listed. Air mass pathways (A–E) are defined in Fig. S1.

| Category | Initial factors | MLR | | | ARIMAX | | |
| --- | --- | --- | --- | --- | --- | --- | --- |
|  |  | Fungi | Protists | Metazoa | Fungi | Protists | Metazoa |
| Thermodynamic variable | Temp.  Humidity  Abs. humidity  Dewpoint  Vapor press.  Surface temp. | Temp.  Humidity  Abs. humidity  Surface temp. | - | Vapor press. | Humidity | Dewpoint | Vapor press. |
| Radiative conditions | Cloud  Solar radiation  Sunshine  Ultraviolet | Cloud  Sunshine  Ultraviolet | - | Cloud Ultraviolet | - | - | Ultraviolet |
| Atmospheric circulation | Wind velocity  Air press.  Retention | Retention  Air press. | Wind velocity  Retention | Air press. | - | - | - |
| Hydrological variables | Precipitation  Evaporation | Precipitation | - | Precipitation  Evaporation | Precipitation, Evaporation | - | Precipitation  Evaporation |
| Oceanic conditions  & Others | Water temp.  Wave height  Wave period  Traffic | Retention  Air press. | - | Water temp.  Wave height | Water temp. Wave period | - | Water temp.  Traffic |
| Air mass transport pathways | A, B, C, D, E | A, D | - | A, D, E | D | A, D | - |


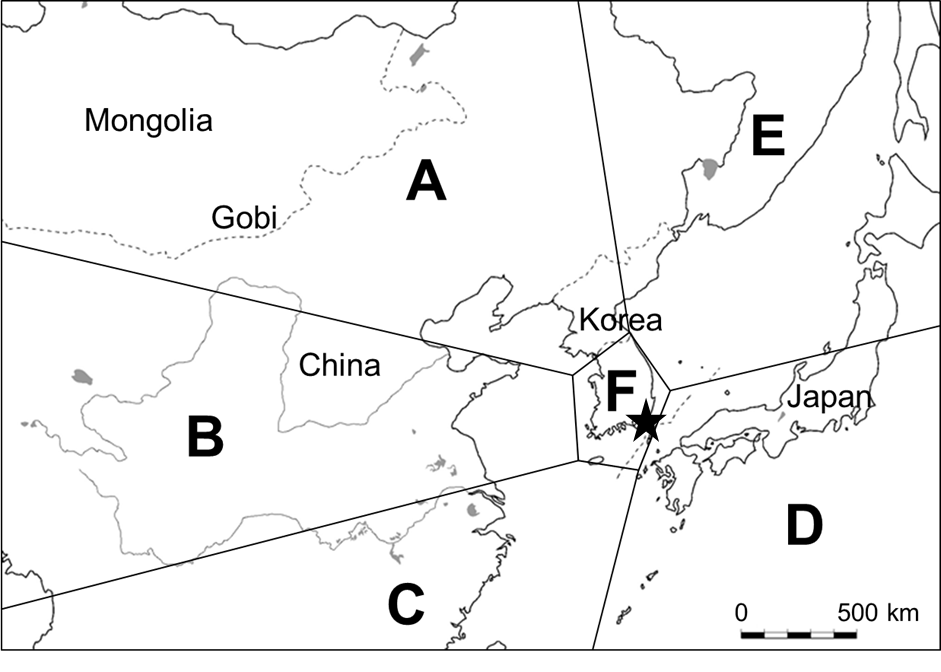
Fig. S1. Classification of air mass transport pathways (trajectories) in this study. Six pathway categories (A–F) were defined based on 72-h backward HYSPLIT trajectories. Pathway A represents air masses originating from northwestern and northern China; B, from mid-western China; C, from southern China; D, from southern Japan; E, from the East Sea and northern Japan; and F, locally retained air masses over the Korean Peninsula. The star indicates the aerosol sampling site in Busan, South Korea.


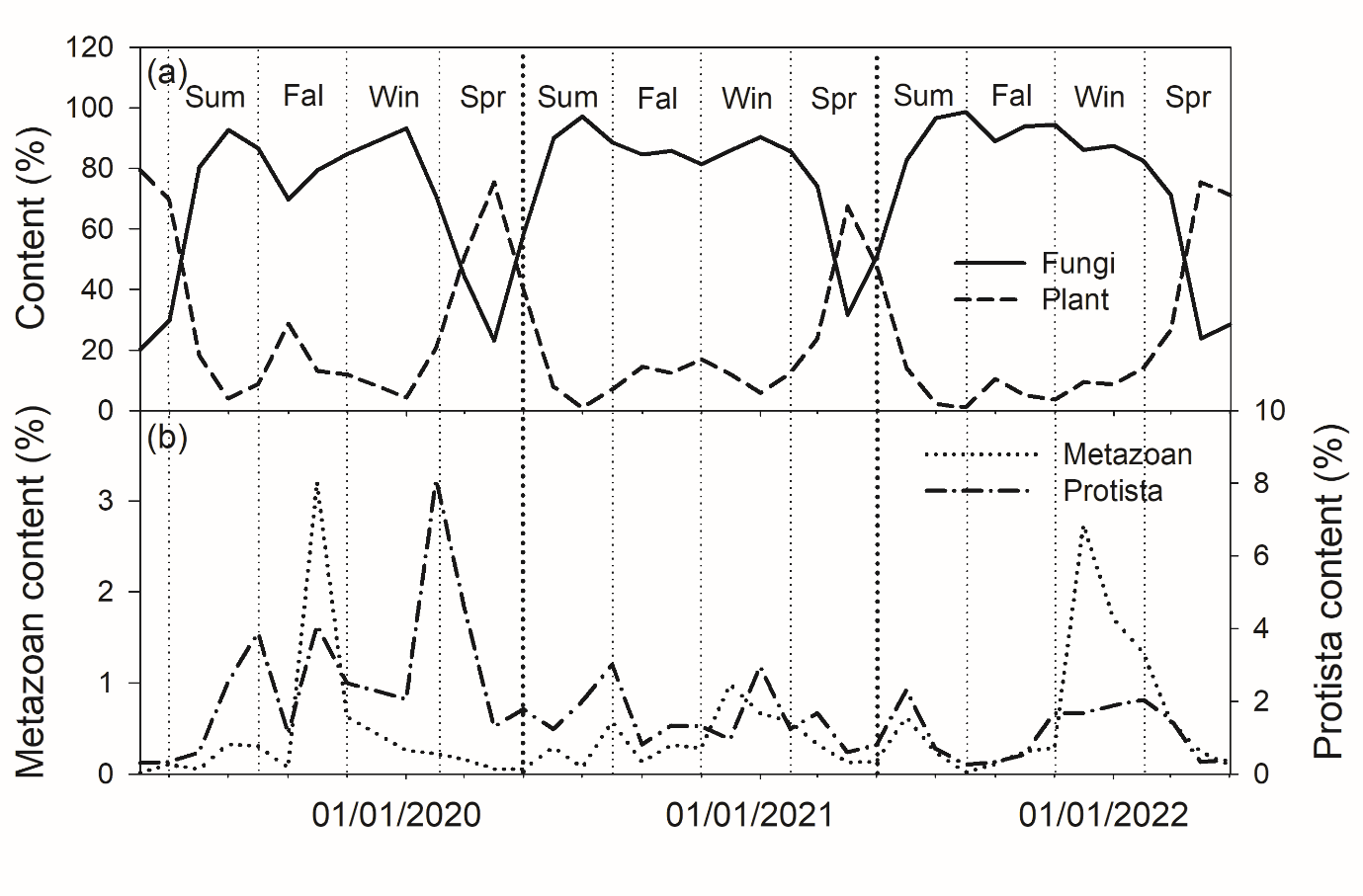


Fig. S2. Temporal changes in the relative abundances (i.e., proportional composition) of major eukaryotic groups in aerosols. (a) This figure shows the fraction of total 18S rRNA gene copies attributable to fungi and plants in each sample over time (April 2019 – May 2022). (b) This panel shows the fraction attributable to metazoa and protists over the same period. Both panels illustrate how the community composition (in percentage terms) varied throughout the study. Vertical dashed lines indicate seasonal transitions, with seasons labeled as Spring (Spr), Summer (Sum), Fall (Fal), and Winter (Win).


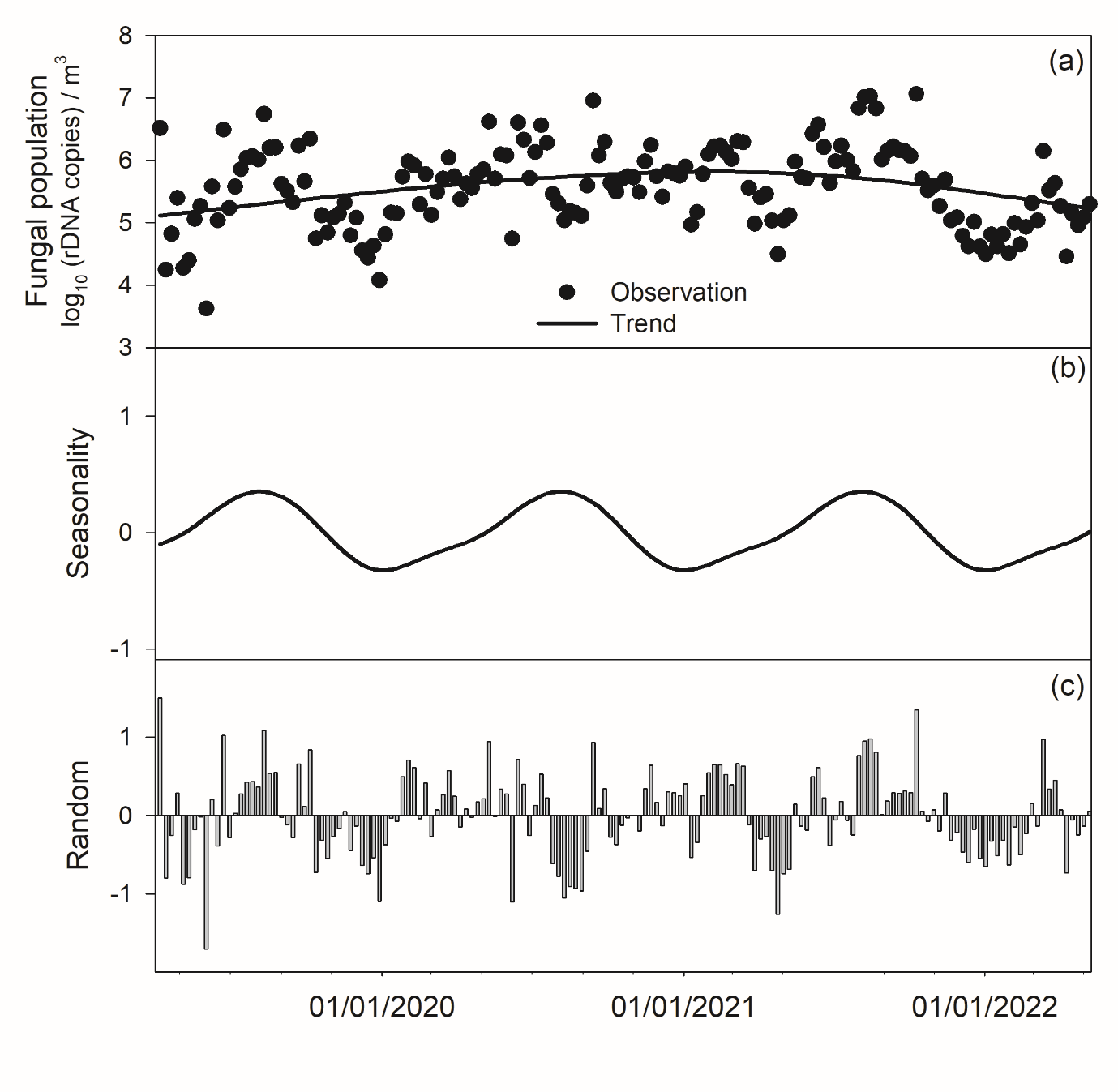


Fig. S3. Time-series decomposition of the fungal population over the three-year study period. Weekly fungal abundance data were decomposed into trend, seasonal, and irregular components using structural time-series analysis, highlighting the underlying seasonal pattern.


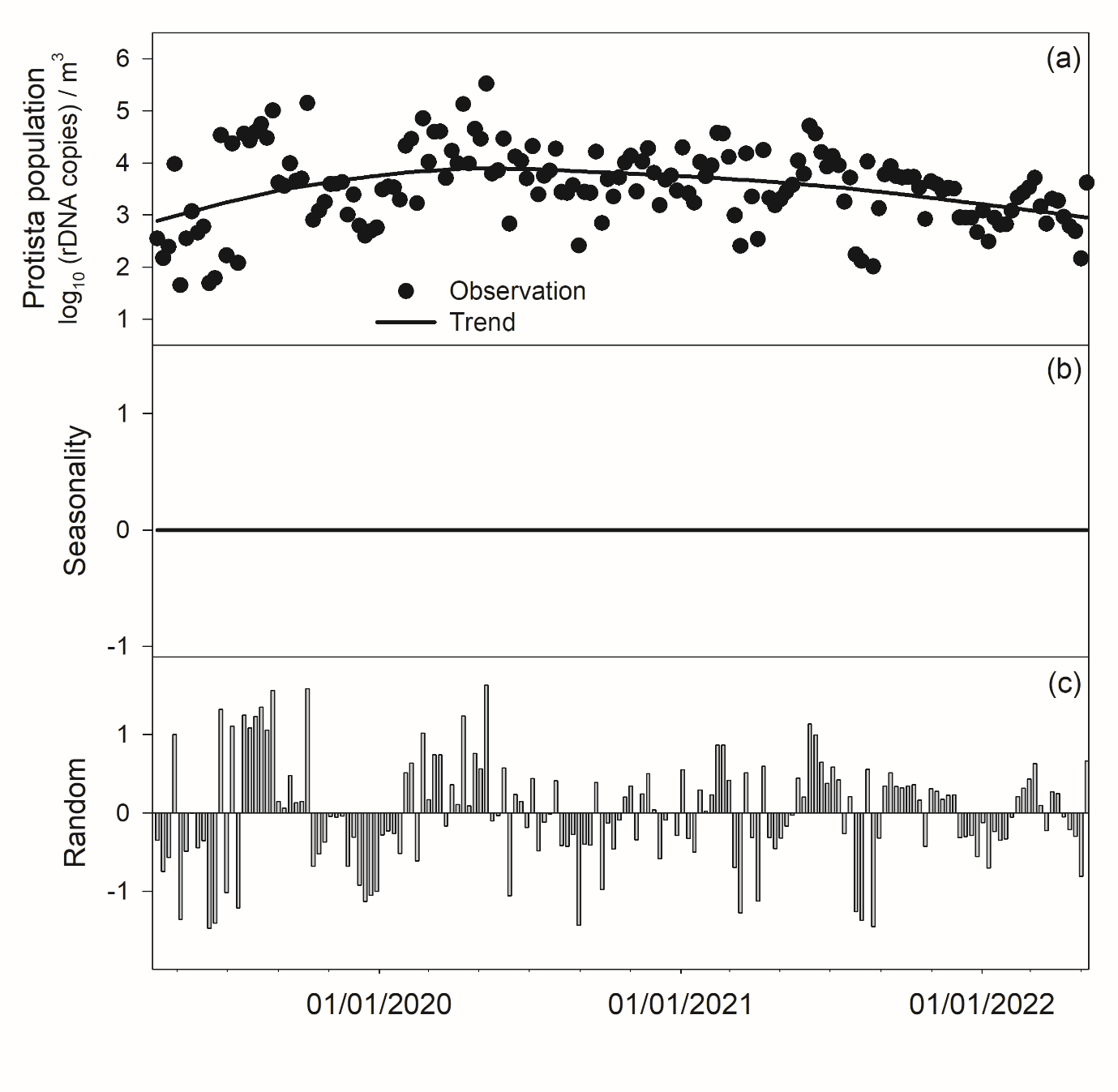


Fig. S4. Time-series decomposition of the protist population over the three-year study period. Weekly protist abundance data were decomposed into trend, seasonal, and irregular components using structural time-series analysis, showing minimal seasonal variation.


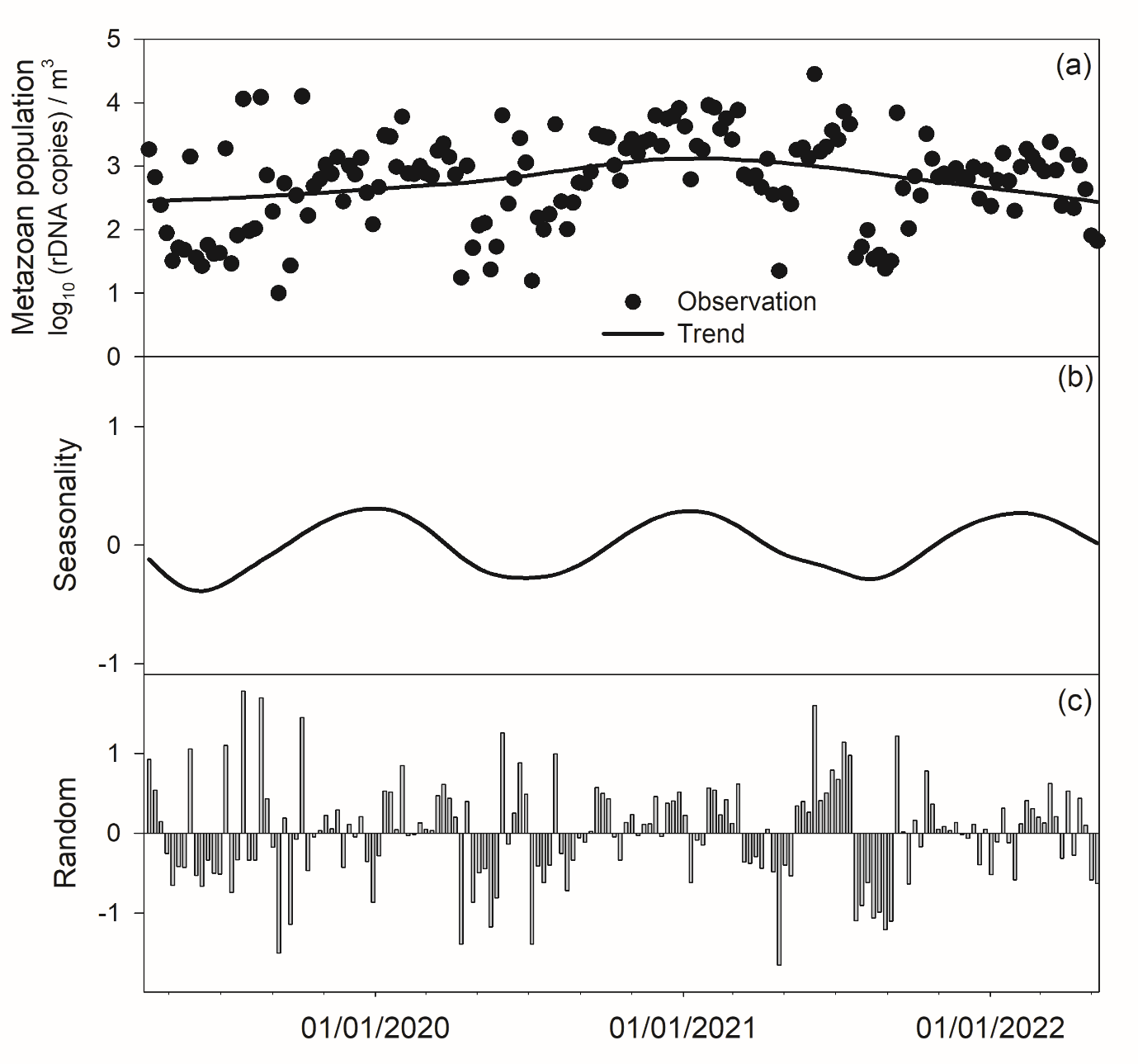


Fig. S5. Time-series decomposition of the metazoan population over the three-year study period. Weekly metazoan abundance data were decomposed into trend, seasonal, and irregular components using structural time-series analysis, illustrating the detected seasonal cycle.


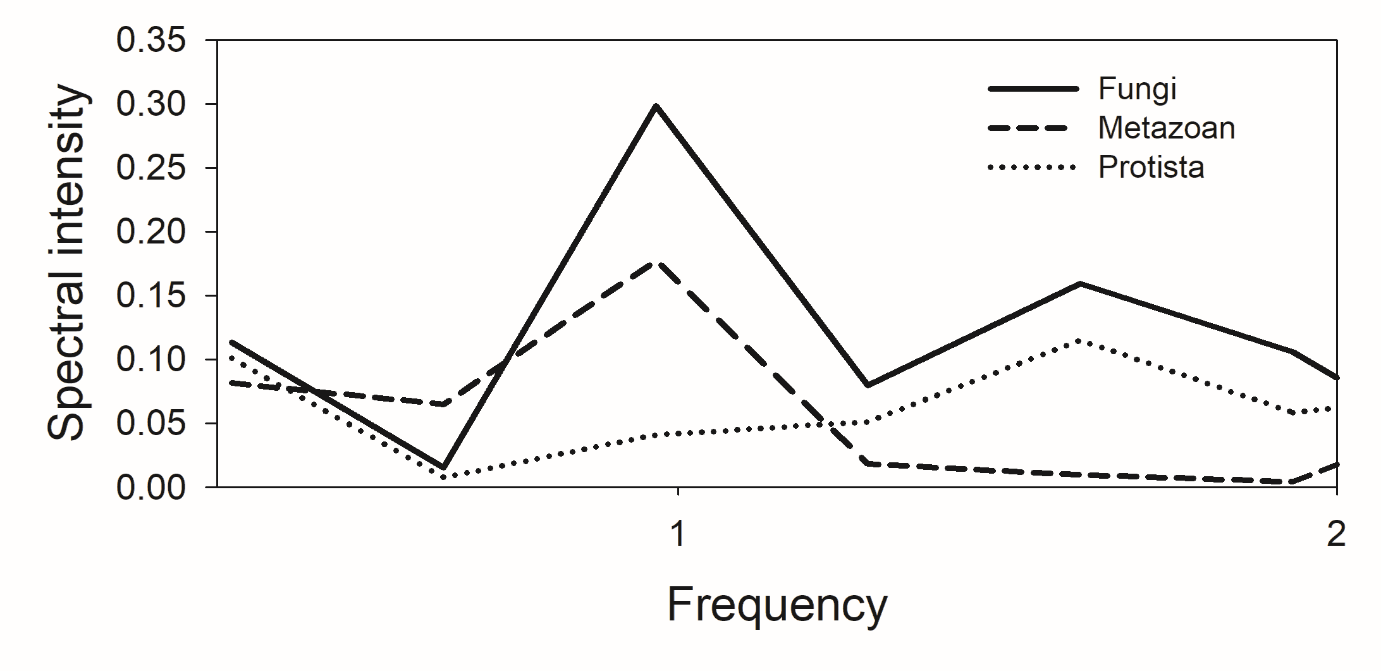


Fig. S6. Spectral analysis of weekly eukaryotic abundance time series to identify seasonal frequency. This figure presents the power spectral density of the time series for fungi, protists, and metazoa.


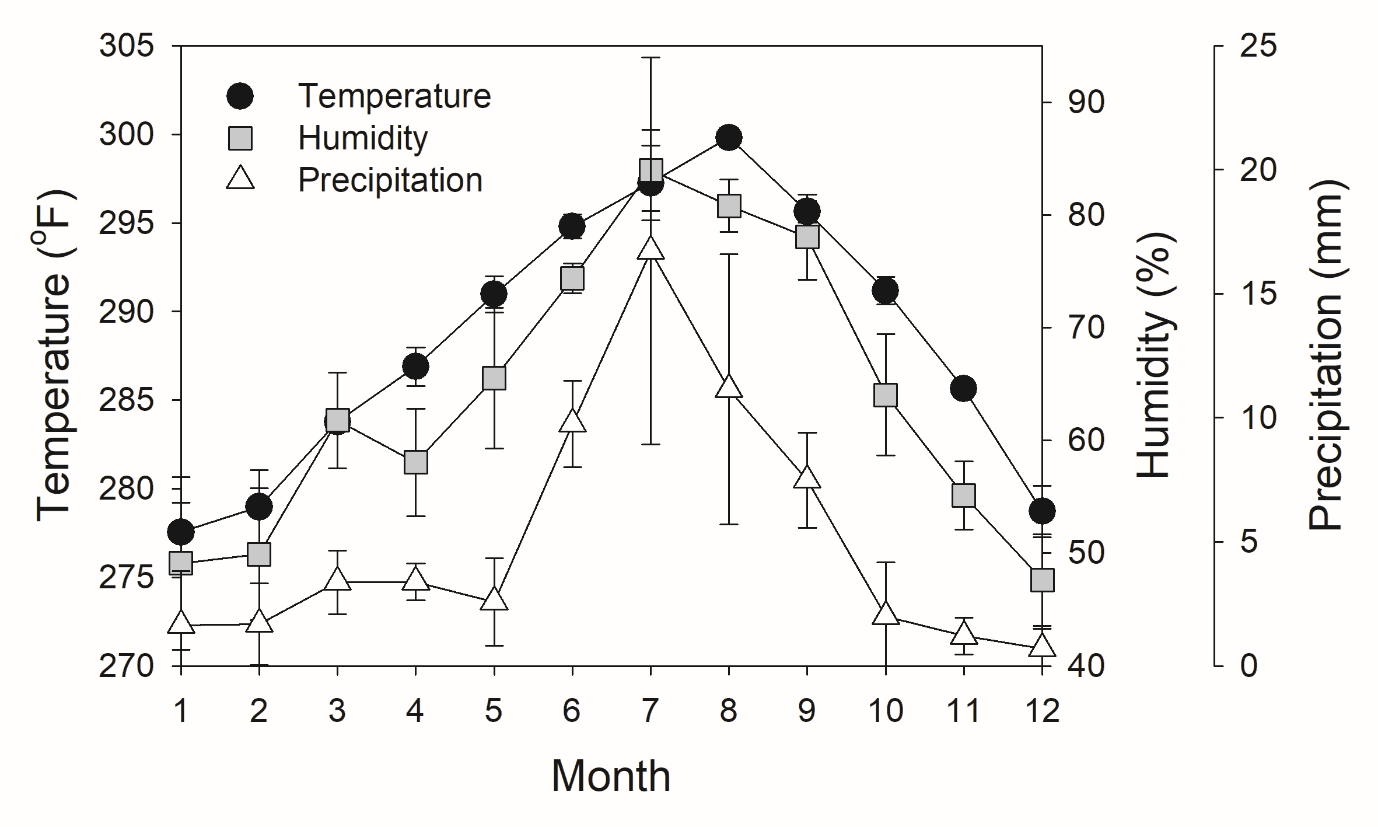


Fig. S7. Seasonal variations in key meteorological factors (I): temperature, humidity, and precipitation. Monthly mean air temperature (°F), relative humidity (%), and total precipitation (mm) are shown for each month, averaged across the three study years. Error bars indicate ±1 standard deviation.


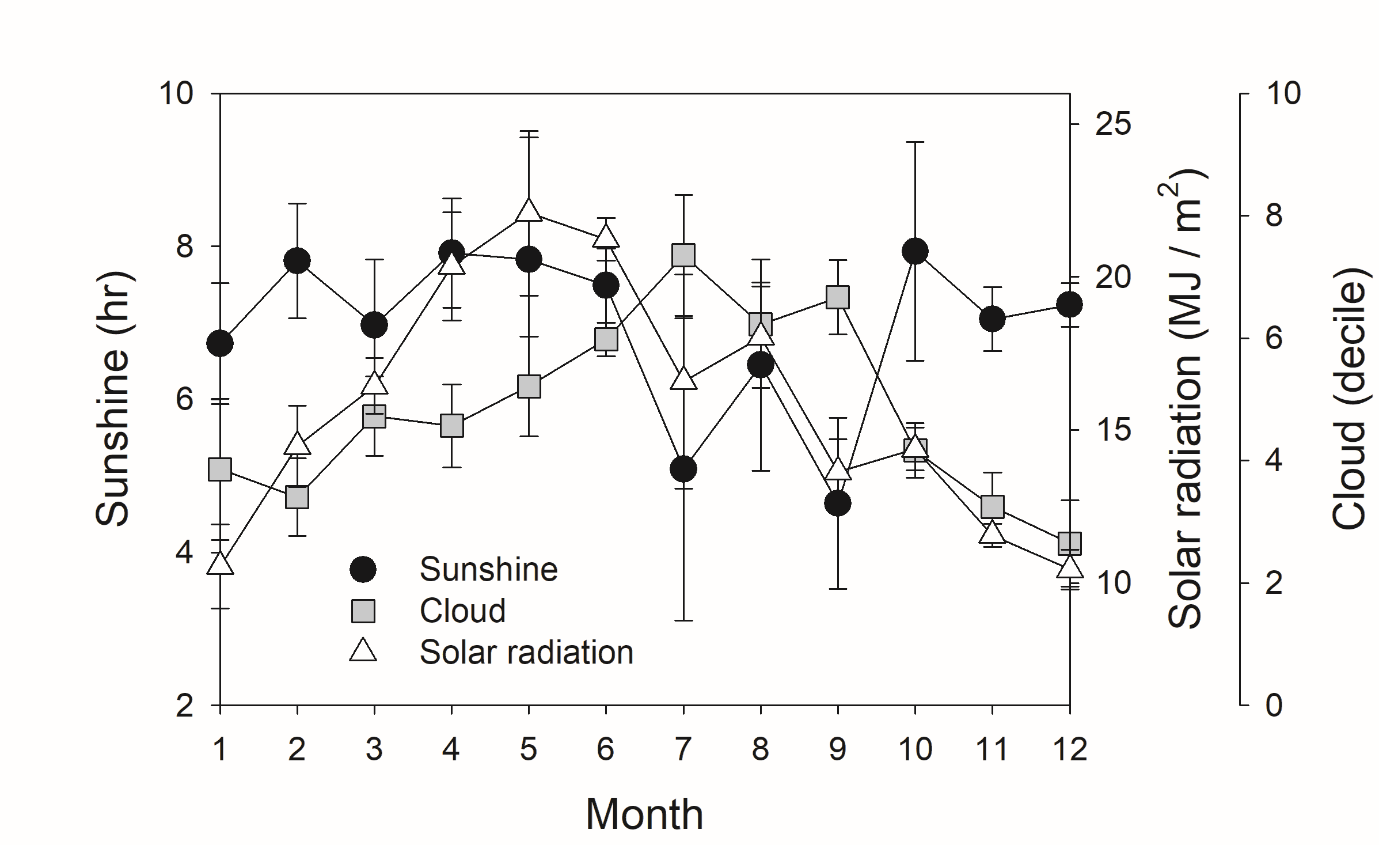


Fig. S8. Seasonal variations in key meteorological factors (II): sunshine duration, solar radiation, and cloud cover. Monthly mean sunshine duration (hr d⁻¹), solar radiation (MJ m⁻²), and cloud cover (decile) are shown for each month, averaged across the three study years. Error bars indicate ±1 standard deviation, illustrating interannual variability.


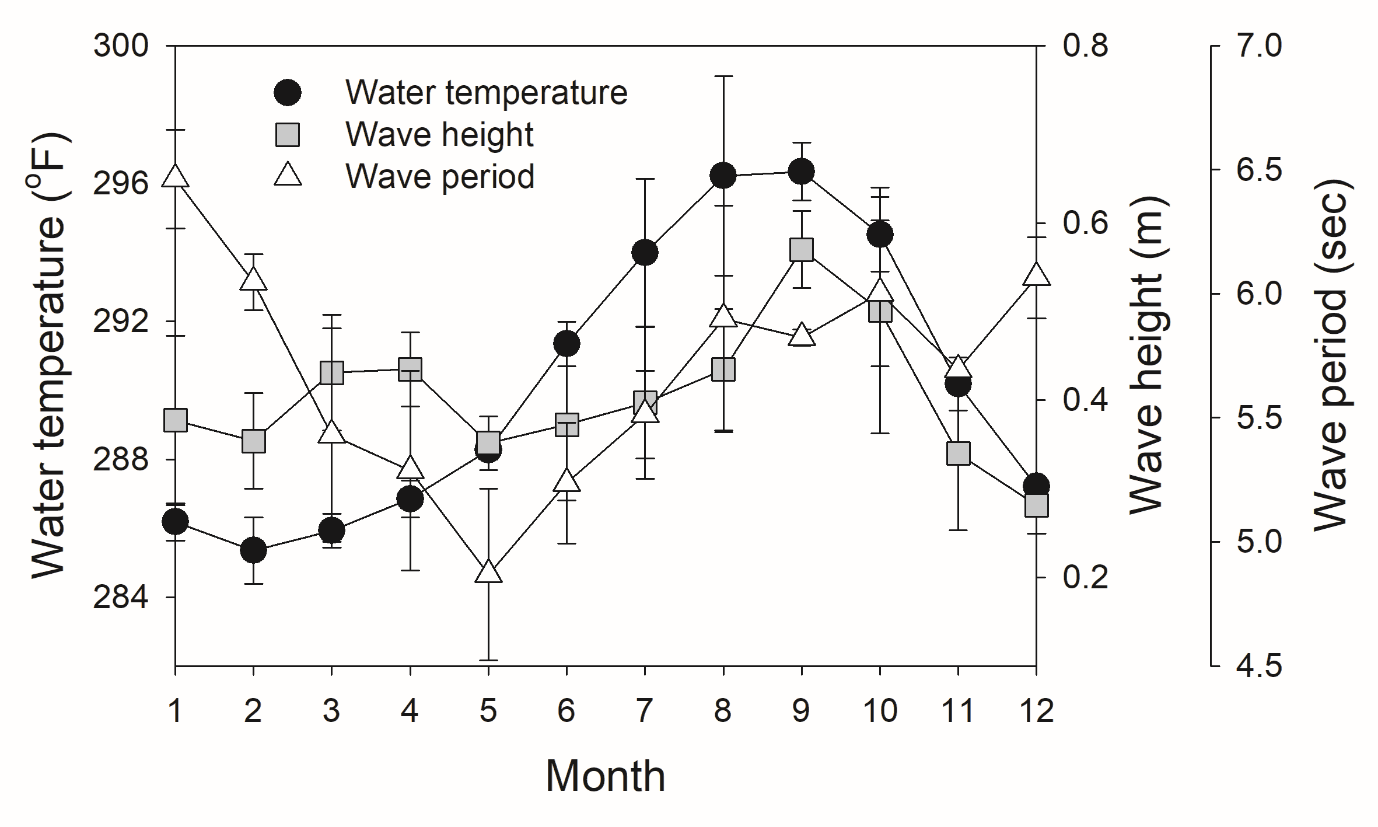


Fig. S9. Seasonal variations in key meteorological factors: coastal and oceanic conditions. Monthly mean sea surface temperature (°F), wave height (m), and wave period (s) are shown for each month, averaged across the three study years from nearby coastal monitoring stations. Error bars indicate ±1 standard deviation, illustrating interannual variability.


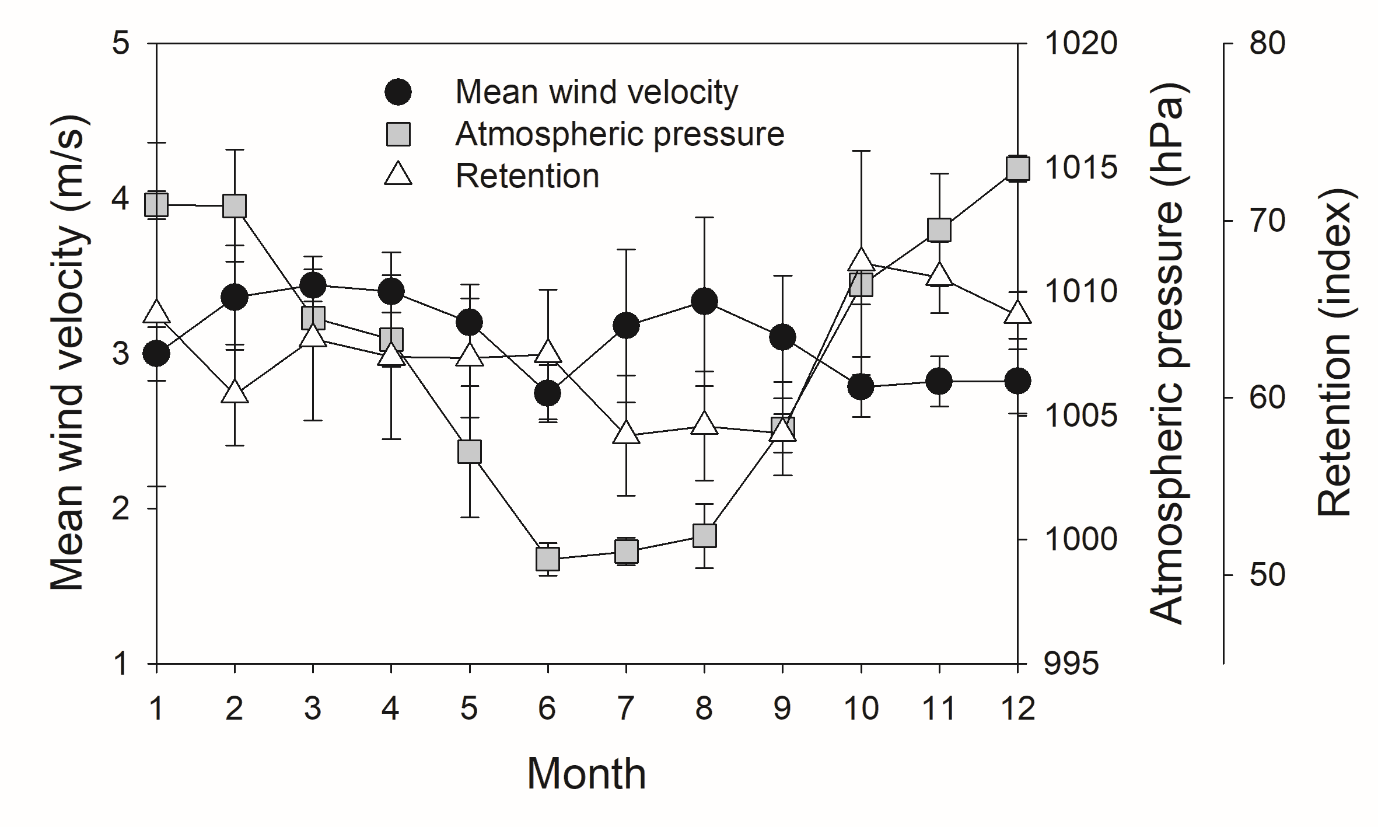


Fig. S10. Seasonal variations in key meteorological factors: wind, pressure, and atmospheric retention. Monthly mean wind speed (m s⁻¹), sea-level atmospheric pressure (hPa), and retention index (a dimensionless indicator of atmospheric stability or stagnation) are shown for each month, averaged across the three study years. Error bars indicate ±1 standard deviation, illustrating interannual variability.
